# Functional Characterization of the Effects of *N*-acetyltransferase 2 Alleles on *N*-acetylation of Eight Drugs and Worldwide Distribution of Substrate-Specific Diversity

**DOI:** 10.3389/fgene.2021.652704

**Published:** 2021-03-18

**Authors:** Koya Fukunaga, Ken Kato, Takuji Okusaka, Takeo Saito, Masashi Ikeda, Teruhiko Yoshida, Hitoshi Zembutsu, Nakao Iwata, Taisei Mushiroda

**Affiliations:** ^1^Laboratory for Pharmacogenomics, RIKEN Center for Integrative Medical Sciences, Yokohama, Japan; ^2^Department of Head and Neck Medical Oncology, National Cancer Center Hospital, Tokyo, Japan; ^3^Department of Hepatobiliary and Pancreatic Oncology, National Cancer Center Hospital, Tokyo, Japan; ^4^Department of Psychiatry, Fujita Health University School of Medicine, Toyoake, Japan; ^5^Fundamental Innovative Oncology Core, National Cancer Center Research Institute, Tokyo, Japan

**Keywords:** dapsone, genetic diversity, isoniazid, slow acetylators, sulfamethazine, sulfapyrizine, *N*-acetyltransferase 2 (NAT2)

## Abstract

Variability in the enzymatic activity of *N*-acetyltransferase 2 (NAT2) is an important contributor to interindividual differences in drug responses. However, there is little information on functional differences in *N*-acetylation activities according to NAT2 phenotypes, i.e., rapid, intermediate, slow, and ultra-slow acetylators, between different substrate drugs. Here, we estimated *NAT2* genotypes in 990 Japanese individuals and compared the frequencies of different genotypes with those of different populations. We then calculated *in vitro* kinetic parameters of four NAT2 alleles (NAT2^∗^4, ^∗^5, ^∗^6, and ^∗^7) for *N*-acetylation of aminoglutethimide, diaminodiphenyl sulfone, hydralazine, isoniazid, phenelzine, procaineamide, sulfamethazine (SMZ), and sulfapyrizine. NAT2^∗^5, ^∗^6, and ^∗^7 exhibited significantly reduced *N*-acetylation activities with lower Vmax and CLint values of all drugs when compared with NAT2^∗^4. Hierarchical clustering analysis revealed that 10 *NAT2* genotypes were categorized into three or four clusters. According to the results of *in vitro* metabolic experiments using SMZ as a substrate, the frequencies of ultra-slow acetylators were calculated to be 29.05–54.27% in Europeans, Africans, and South East Asians, whereas Japanese and East Asian populations showed lower frequencies (4.75 and 11.11%, respectively). Our findings will be helpful for prediction of responses to drugs primarily metabolized by NAT2.

## Introduction

*N*-acetyltransferase 2 (NAT2) is responsible for *N*-acetylation of aromatic amines and hydrazine derivatives. Signature single nucleotide polymorphisms (SNPs) for each haplotype cluster of the *NAT2* gene in the Human Arylamine *N*-acetyltransferase Gene Nomenclature^1^, particularly c.191G > A (rs1801279; *NAT2*^∗^14), c.341T >C (rs1801280; *NAT2*^∗^5), c.590G > A (rs1799930; *NAT2*^∗^6), and c.857G > A (rs1799931; *NAT2*^∗^7), are common observed among 128 populations (Sabbagh et al., 2011). *NAT2^∗^4* is reported as the wild-type allele, whereas *NAT2^∗^5*, *^∗^6*, and *^∗^7* have nucleotide substitutions and result in decreased function (Hein et al., 2008). Individuals with each genotype are classified into three groups, i.e., rapid acetylators (RAs), intermediate acetylators (IAs), and slow acetylators (SAs), based on the number of RA alleles (i.e., *NAT2^∗^4*) (Birch Kristensen et al., 2018).

NAT2 metabolizes isoniazid (INH), a first-line antituberculosis (TB) drug, to *N*-acetyl INH (Mthiyane et al., 2020). High plasma levels of hydrazine, generated by non-enzymatic conversion of INH, are thought to cause anti-TB drug-induced liver injury (ATDILI) (An et al., 2018; Brewer et al., 2019). Because patients with TB harboring *NAT2^∗^5*, *^∗^6*, and *^∗^7* show much slower *N*-acetylation than patients homozygous for *NAT2^∗^4* (Mthiyane et al., 2020), accumulation of INH and hydrazine occurs in SAs, leading to a higher risk of liver injury (An et al., 2018; Brewer et al., 2019). These phenotypes may be the most efficient pharmacogenomics biomarkers for predicting the risk of ATDILI (Azuma et al., 2013; Mushiroda et al., 2016; Wattanapokayakit et al., 2016; Yuliwulandari et al., 2016; Suvichapanich et al., 2019) and may lead to identification of a cost-effective treatment for TB (Rens et al., 2020). Therefore, categorization of patients as RAs, IAs, and SAs may be useful for treatment with NAT2 substrates (Rens et al., 2020). Although many studies have reported strong associations of each *NAT2* genotype, such as *NAT2^∗^6A/^∗^6A*, with the risk of ATDILI (Suvichapanich et al., 2018; Nicoletti et al., 2020; Yuliwulandari et al., 2021), little information is available regarding the associations between *NAT2* genotype and the risk of the adverse reactions induced by other NAT2 substrate drugs. For example, NAT2 phenotypes, such as SA, IA, and RA, have been shown to be associated with hydralazine-induced adverse reactions because hydralazine is primarily metabolized by NAT2 (Spinasse et al., 2014; Rens et al., 2020). Thus, the substrate specificity of *N*-acetylation with relation to categorization of *NAT2* genotypes into phenotypes has also not been reported (Zang et al., 2007).

As first reported by Ruiz et al. (2012), ultra-slow acetylators (USAs; also known as very-slow acetylators) are defined as individuals with the *NAT2^∗^6A/^∗^6A* genotype. Thereafter, Selinski et al. (2017) reported the existence of USAs based on an association with urinary bladder cancer risk. In addition, we previously demonstrated the existence of USAs (*NAT2^∗^6A/^∗^6A*, *^∗^6A/^∗^7B*, and *^∗^7B/^∗^7B*) by comparing the effects of each *NAT2* genotype on the risk of developing liver injury induced by isoniazid in a trans-ethnic meta-analysis (Suvichapanich et al., 2018). Recently, studies of Indian and European cohorts also concluded that the metabolic effects of NAT2^∗^6 and ^∗^7 are different from that of NAT2^∗^5 (Nicoletti et al., 2020). Therefore, we attempted to clarify the substrate-specific diversity of NAT2 phenotypes between different populations by categorizing *NAT2* genotypes into USA, SA, IA, and RA phenotypes based on the results of *in vitro N*-acetylation of eight drugs in this study.

In this study, we conducted *in vitro* metabolic experiments of aminoglutethimide (AGT), diaminodiphenyl sulfone (DDP), hydralazine (HLZ), isoniazid (INH), phenelzine (PZ), procaineamide (PA), sulfamethazine (SMZ), and sulfapyrizine (SP) using HEK293 cells transiently expressing NAT2^∗^4, ^∗^5, ^∗^6, and ^∗^7 to elucidate the substrate specificity profiles of the *N*-acetylation of each allele. Additionally, we categorized *NAT2* genotypes into phenotypes, i.e., RAs, IAs, SAs, and USAs, based on activity scores calculated by *in vitro* intrinsic clearance (CLint) for *N*-acetylation of the substrate drugs. Moreover, many studies have demonstrated the frequencies of NAT2 phenotypes, but not genotypes, without considering the effects of each drug on *N*-acetylation activity in worldwide populations (Li et al., 2011; Mortensen et al., 2011; Sabbagh et al., 2011). Therefore, we could not categorize *NAT2* genotypes into USA, SA, IA, and RA phenotypes based on the effects of different drugs on *in vitro N*-acetylation activities. Accordingly, we also summarized the worldwide distributions of NAT2 phenotypes in a large-scale study of Japanese individuals and 26 different populations collected by the 1000 Genomes Project (1KGP) based on information on categorization of NAT2 phenotypes.

## Materials and Methods

### Participants and Data Collection

Nine hundred ninety Japanese individuals (343 patients with epilepsy or bipolar disorder, 454 patients with schizophrenia, 65 patients with breast cancer, 83 patients with colorectal cancer, and 45 patients with malignant melanoma) provided informed consent for participation in this study in accordance with the Declaration of Helsinki. The study was approved by the ethics committee of National Cancer Center Research Institute, Fujita Health University Hospital, and RIKEN Center for Integrative Medical Sciences. Targeted resequencing of 100 pharmacokinetics-related genes, including *NAT2*, was performed as reported elsewhere (Fukunaga et al., 2020). Based on the information on *NAT2* SNVs, we estimated individual *NAT2* genotypes and registered this information in the NBDC Human Database^2^. We also obtained individual genotype data for 2,504 samples from 26 ethnic populations collected by the 1KGP^3^. These datasets consisted of high-coverage whole-genome and whole-exome sequencing data from diverse ethnic groups. Using individual genotypes in these datasets, *NAT2* genotypes were determined. The individual genomes from 26 ethnic populations were divided into five major ethnic populations, i.e., Africans (AFRs), Ad mixed Americans (AMRs), East Asians (EASs), Europeans (EURs), and South Asians (SASs). The AFR population consisted of African Caribbeans in Barbados (ACB); Americans of African ancestry in the southwest United States (ASW); Esan in Nigeria (ESN); Luhya in Webuye, Kenya (LWK); Mandinka in the Gambia (MAG); Mende in Sierra Leone (MSL); and Yoruba in Ibadan, Nigeria (YRI). AMR population consisted of Colombians from Medellin, Colombia (CLM); Mexicans from Los Angeles, United States (MXL); Peruvians from Lima, Peru (PEL); and Puerto Ricans from Puerto Rico (PUR). The EAS population consisted of Chinese Dai in Xishuangbanna, China (CDX); Han Chinese in Beijing, China (CHB); Southern Han Chinese (CHS); Japanese in Tokyo, Japan (JPT); and Kinh in Ho Chi Minh City, Vietnam (KHV). The EUR population consisted of Utah residents with northern and western European Ancestry (CEU), Finnish in Finland (FIN), British in England and Scotland (GBR), Iberians in Spain (IBS), and Toscani in Italy. The SAS population consisted of Bengali from Bangladesh (BEB); Gujarati Indians from Houston, TX, United States (GIH); Indian Telugu from the United Kingdom (ITU); Punjabis from Lahore, Pakistan (PJL); and Sri Lankan Tamils from the United Kingdom (STU).

### Expression of *NAT2^∗^4, ^∗^5, ^∗^6, and ^∗^7*

**FIGURE 1:**
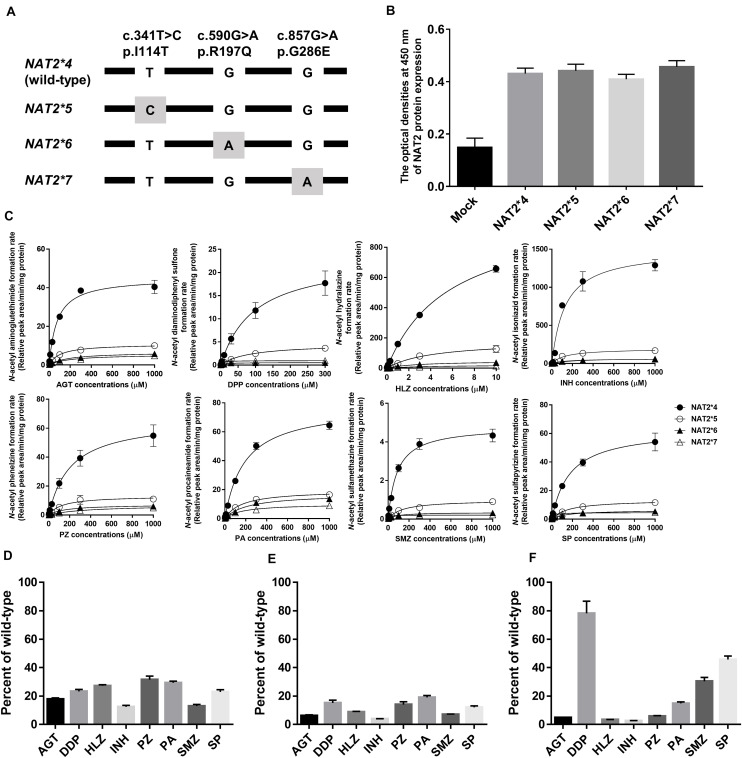
*N*-Acetylation of eight drugs by *NAT2* alleles. **(A)** Schematic of *NAT2* alleles. The gray box represents signature SNVs. The nucleotide and amino acid positions are based on NM_000015.2 RefSeq. **(B)** NAT2 protein expression levels in lysates from HEK293 cells expressing NAT2^∗^4, ^∗^5, ^∗^6, and ^∗^7 alleles, as determined by ELISA. The *Y* axis represents the optical density at 450 nm. Each value represents the mean + SEM of four independent experiments. **(C)** Michaelis-Menten curves of the enzymatic activities of NAT2 proteins encoded by *NAT2^∗^4*, *^∗^5*, *^∗^6*, and *^∗^7* alleles toward eight drugs (aminoglutethimide, AGT; diaminodiphenyl sulfone, DDP; hydralazine, HLZ; isoniazid, INH; phenelzine, PZ; procaineamide, PA; sulfamethazine, SMZ; and sulfapyrizine, SP). Each point represents the mean ± standard deviation of four determinations. Open rhombus, closed rhombus, open triangle, and closed triangle present NAT2^∗^4, ^∗^5, ^∗^6, and ^∗^7, respectively. **(D–F)** Relative CLint values of NAT2^∗^5 **(D)**, ^∗^6 **(E)**, and ^∗^7 **(F)** among eight drugs using the CLint value of NAT2^∗^4 as a base of 100. Each value represents the mean + SEM of four independent experiments.

The cDNAs of *NAT2^∗^4*, *^∗^5*, *^∗^6*, and *^∗^7* were synthesized by Integrated DNA Technologies (Coralville, IA, United States) and cloned into the *Eco*RV site of pcDNA3.1 (+) Mammalian Expression Vectors (Thermo Fisher Scientific, Waltham, MA, United States) using an In-Fusion HD Cloning Kit (Takara, Shiga, Japan). The locations of variants in the *NAT2* alleles are shown in Figure 1A. The constructs were transformed into Escherichia coli JM109 competent cells (Takara) and then the sequences of the inserts in a few colonies were confirmed by Sanger sequencing. After obtaining the constructs carrying each allele, the constructs were cloned and purified with a Qiagen Midi Plasmid Kit (Qiagen, Valencia, CA, United States). The sequences of the clones carrying each allele were confirmed by Sanger sequencing again. The concentration and quality of DNA were determined using a Nano Drop 1000 UV-Vis Spectrophotometer (Thermo Fisher Scientific).

HEK293 cells were seeded into 10-cm collagen-coated culture dishes (IWAKI, Tokyo, Japan) in Dulbecco’s modified Eagle’s medium (Sigma-Aldrich, St. Louis, MO, United States) containing 10% fetal bovine serum (FBS; Sigma-Aldrich), 100 mM sodium pyruvate (Thermo Fisher Scientific), and non-essential amino acid solution (Thermo Fisher Scientific). When the cells were approximately 80% confluent, vectors carrying each allele were transfected into the cells using Lipofectamine 3000 Transfection Reagent (Thermo Fisher Scientific) according to the manufacturer’s instructions. The optimal transfection efficiency and cell viability were obtained with 10 μg DNA/dish and 30 μL Lipofectamine 3000 Transfection Reagent. Forty-eight hours after transfection, cells were treated with 0.05% trypsin-ethylenediaminetetraacetic acid (EDTA; Thermo Fisher Scientific) and washed once with 100 mM potassium phosphate buffer (pH 7.4). The washed cell pellet was then lysed with occasional mixing in 1 mL of Mammalian Protein Extraction Buffer (GE Healthcare, Waukesha, WI, United States) containing EDTA-free protease inhibitor cocktail (Takara). The homogenate was centrifuged at 20,000 × *g* and 4°C for 30 min. The resulting supernatant was transferred to another tube and stored at −80°C until subsequent analysis.

### Measurement of *NAT2* Protein Expression Levels

Total protein concentrations of lysates were measured using a Pierce BCA protein assay kit (Thermo Fisher Scientific) according to the manufacturer’s instructions. Equal amounts of protein were used for enzyme-linked immunosorbent assay (ELISA). The lysates (0.5 mg protein) were coated onto 96-well microplates for 20 h at 4°C. After washing the plates three times by filling the wells with 200 μL phosphate-buffered saline (PBS), the remaining protein-binding sites in the coated wells were blocked by adding 200 μL of 5% FBS (Merck, Darmstadt, Germany) in PBS. After the plates were washed three times, 100 μL of primary anti-NAT2 monoclonal antibody (1:100; cat. no. sc-134399; Santa Cruz Biotechnologies, Dallas, TX, United States) was added to each well, and plates were then incubated for 1 h. After washing three times, 100 μL of HRP-Rabbit Anti-Mouse IgG (H + L) Conjugate (1:100,000; Thermo Fisher Scientific) was added to each well, and plates were incubated for 30 min. After washing three times, we added 100 μL of an ELISA POD substrate TMB kit (Nacalai Tesque, Kyoto, Japan) to each well and incubated the plates for 10 min. An equal volume of 1 M HCl as stopping solution was added, and the optical density at 450 nm was measured using a microplate reader (ARVOmx; PerkinElmer, Waltham, MA, United States).

### *N*-Acetylation of Eight Drugs

Lysates containing 0.01 mg/mL protein, 50 mM triethanolamine (pH 7.5), 1 mM EDTA, 1 mM dithiothreitol, 1.08 mg/ml acetyl-D, L-carnitine, 0.22 U/mL carnitine acetyltransferase, and 0.1 mM acetyl coenzyme were incubated at 37°C with the substrates of the different concentrations (AGT; 0, 0.3, 1, 3, 10, 30, 100, 300, or 1000 μM, DPP; 0, 0.1, 0.3, 1, 3, 10, 30, 100, or 300 μM, HLZ; 0, 0.003, 0.01, 0.03, 0.1, 0.3, 1, 3, 10 μM, INH; 0, 0.3, 1, 3, 10, 30, 100, 300, or 1000 μM, PZ; 0, 0.3, 1, 3, 10, 30, 100, 300, or 1000 μM, PA; 0, 0.3, 1, 3, 10, 30, 100, 300, or 1000 μM, SMZ; 0, 0.3, 1, 3, 10, 30, 100, 300, or 1000 μM, SP; 0, 0.3, 1, 3, 10, 30, 100, 300, or 1000 μM). The total volume of all incubations was 100 μL. After incubation for 20 min with gentle shaking, reactions were quenched with 100 μL cold acetonitrile containing 100 μM ticlopidine (Thermo Fisher Scientific) as an internal standard. The mixture was centrifuged at 20,000 × *g* and 4°C for 5 min, and 2 μL of the supernatant was injected for ultra-high-performance liquid chromatography-tandem mass spectrometry (UPLC-MS/MS) analysis. All reactions were performed in triplicate independently.

UPLC-MS/MS analysis was performed on a Waters ACQUITY UPLC system coupled to a TQ Detector (Waters, Milford, MA, United States). Chromatographic separation was achieved on an ACQUITY UPLC BEH Amide column (2.1 mm × 150 mm, 1.7 μm; Waters) equipped with a Vanguard pre-column (ACQUITY UPLC BEH Amide, 2.1 mm × 5 mm, 1.7 μm; Waters). The column temperature was kept at 45°C, and samples in the autosampler were maintained at 7°C. The mobile phases (flow rate: 0.4 mL/min) were 0.1% formic acid (FA) in water/acetonitrile (20:80, v/v), isopropanol (IPA)/0.1% FA/acetonitrile (1:1:98, v/v/v), 0.1% FA/acetonitrile (10:90, v/v), 0.1% FA/acetonitrile (30:70, v/v), IPA/acetonitrile (80:20, v/v), 0.1% FA/acetonitrile (90:10, v/v), 0.1% FA/acetonitrile (10:90, v/v), and 0.1% FA/acetonitrile (20:80, v/v) for AGT, DDP, HLZ, INH, PZ, PA, SMZ, and SP, respectively. Multiple reaction monitoring (MRM) in the positive ion mode was performed on *m/z* 275→230, *m/z* 291→156, *m/z* 203→89, *m/z* 180→121, *m/z* 179→105, *m/z* 278→205, *m/z* 321→186, *m/z* 292→134, and *m/z* 123.5→79 for acetyl AGT, acetyl DDP, acetyl HLZ, acetyl INH, acetyl PZ, acetyl PA, acetyl SMZ, acetyl SP, and the internal standard, respectively. Universal mass spectrometric settings included capillary voltage of 2.0 kV, cone voltage of 30 V, extractor voltage of 3 V, RF Lens of 0.1, source temperature of 120°C, desolvation temperature of 500°C, desolvation gas flow of 500 L/h, cone gas flow of 50 L/h, collision energy of 20 V, and dwell time of 100 ms. MRM peak integrations and data analyses were performed using MassLynx 4.1 (Waters). The total run time of the analyses was 3 min. The linearity of the assay for each metabolite was confirmed using serial dilutions of the positive control sample after a 60-min incubation with NAT2^∗^4. Since no authentic standards for *N*-acetyl conjugates of all substrates were commercially available, the relative peak area based on the ratio of the analyte signal for the internal standard were used to measure metabolite levels. We determined the limit of detection (LOD) based on the analyte peaks with a signal-to-noise (S/N) ratio of 10 for each *N*-acetyl conjugation. The values of the S/N ratio were 145.8, 180.5, 167.6, 90.3, 10.1, 94.7, 104.3, and 36.5 for acetyl AGT, acetyl DDP, acetyl HLZ, acetyl INH, acetyl PZ, acetyl PA, acetyl SMZ, and acetyl SP, respectively, when the *in vitro* metabolic studies were conducted using the minimum substrate concentration.

### Statistics

Haplotype analysis of *NAT2* was performed using SNPAlyze software (version 8.0.1; Dynacom, Chiba, Japan). Using Graph Pad Prism software (version 6; GraphPad Software, San Diego, CA, United States), each data set was individually fitted to the Michaelis–Menten equation and the kinetic parameters (Km and Vmax) were determined by the Lineweaver-Burk plots. Protein expression levels and kinetic data were presented as means + or ± standard errors (SEs) of four independent experiments and were analyzed by Tukey’s multiple comparison tests. Results with *P* values less than 0.05 were considered statistically significant. Ward’s D2 method and Euclidean distances of the hierarchical agglomerative clustering were used to classify *NAT2* genotypes based on the CLint value of each drug. All calculations except for kinetic parameters and visualization of the calculated values were performed using R software (version 3.5.0; R Foundation for Statistical Computing, Vienna, Austria). The most optimal numbers of clusters showing the highest values by NbClust R-packages were adapted, and the grouping of each *NAT2* genotype was determined by visual observation of a dendrogram for each drug.

## Results

A partial schematic diagram of *NAT2* alleles is shown in Figure 1A. *NAT2* genotypes in 990 Japanese individuals were estimated based on information on individual SNVs (Table 1). To compare the genetic diversity in the frequencies of *NAT2* genotypes, we also estimated *NAT2* genotypes in AFR (*N* = 661), AMR (*N* = 347), EAS (*N* = 504), EUR (*N* = 503), and SAS (*N* = 489) populations collected by the 1KGP; 10 genotypes were identified (Table 1). The genotype frequencies of *NAT2^∗^4/^∗^4* in Japanese, AFR, AMR, EAS, EUR, and SAS populations were 48.5, 20.3, 14.4, 31.2, 6.8, and 5.9%, respectively. The frequencies of RAs in EAS populations, including the Japanese population, were higher than those in EUR and SAS populations, supporting the large ethnic differences in NAT2 phenotype frequencies. The detailed genotype frequencies are shown in Supplementary Table 1. The highest and lowest frequencies of *NAT2^∗^4* allele in seven African subpopulations were 0.523 of YRI and 0.352 of ASW, respectively. This indicates that the sums of frequencies of *NAT2^∗^5*, *^∗^6*, and *^∗^7* alleles fin YRI and ASW populations were lowest (0.473) and highest (0.648), respectively. In three Chinese subpopulations (CDX, CHB, and CHS), the *NAT2^∗^4* allele frequencies showed a marked difference (0.457, 0.607, and 0.524) as well as the African subpopulations.

**TABLE 1:** Frequency of *NAT2* genotypes in 990 Japanese individuals and 2,504 individuals from five populations collected by the 1KGP.

	Japanese^1^		AFR^2^		AMR^2^		EAS^2^		EUR^2^		SAS^2^	
Genotypes	Number	Frequency	Number	Frequency	Number	Frequency	Number	Frequency	Number	Frequency	Number	Frequency
*4/*4	480	0.485	134	0.203	50	0.144	157	0.312	34	0.068	29	0.059
*4/*5	23	0.023	163	0.247	84	0.242	16	0.032	107	0.213	75	0.153
*4/*6	260	0.263	134	0.203	33	0.095	115	0.228	66	0.131	74	0.151
*4/*7	125	0.126	19	0.029	29	0.084	86	0.171	6	0.012	13	0.027
*5/*5	0	0	62	0.094	52	0.150	1	0.002	108	0.215	63	0.129
*5/*6	5	0.005	92	0.139	46	0.133	15	0.030	117	0.233	117	0.239
*5/*7	2	0.002	7	0.011	17	0.049	5	0.010	12	0.024	21	0.043
*6/*6	42	0.042	38	0.057	13	0.037	40	0.079	48	0.095	65	0.133
*6/*7	44	0.044	12	0.018	14	0.040	48	0.095	5	0.010	31	0.063
*7/*7	9	0.009	0	0	9	0.026	21	0.042	0	0	1	0.002

*AFR, African; AMR, Ad mixed American; EAS, East Asian; EUR, European; SAS, South Asian; 1KGP, 1000 Genomes Project. ^1^The data were deposited in the National Bioscience Database Center (NBDC) Human Database (https://humandbs.biosciencedbc.jp/en/hum0163-v2). ^2^The data were obtained from http://ftp.1000genomes.ebi.ac.uk/vol1/ftp/release/20130502/.*

Recombinant NAT2 proteins were transiently expressed in HEK293 cells, and the lysates were used for *in vitro* metabolic studies. As shown in Figure 1B, all NAT2 proteins were immunodetectable using an anti-NAT2 monoclonal antibody, and there were no differences in the expression levels of recombinant proteins. The catalytic activities of NAT2^∗^4, ^∗^5, ^∗^6, and ^∗^7 proteins were evaluated using the eight substrate drugs. Michaelis-Menten plots of the four NAT2 proteins are shown in Figure 1C, and the estimated kinetic parameters (Km, Vmax, and CLint) are summarized in Table 2. The three variant proteins, i.e., NAT2^∗^5, ^∗^6, and ^∗^7, exhibited significantly reduced Vmax (2.20-25.09% that of NAT2^∗^4) and CLint (2.62-72.40% that of NAT2^∗^4) values for all drugs. Although most Km values for the variant proteins were comparable to or higher than that of NAT2^∗^4, NAT2^∗^7 showed significantly lower Km values in the *N*-acetylation of DDP (5.3% that of NAT2^∗^4), SMZ (14.3% that of NAT2^∗^4), and SP (15.7% that of NAT2^∗^4) compared with NAT2^∗^4 (Table 2). When the CLint values of NAT2^∗^4 were set at 100%, the relative clearance values of NAT2^∗^5, ^∗^6, and ^∗^7 were 12.4-30.0%, 3.9-18.7%, and 2.6-70.2%, respectively (Figures 1D–F). The *P* values for differences in relative clearance between the eight drugs are summarized in Supplementary Table 2. When comparing the relative clearance value of NAT2^∗^7 for each drug, the values for DDP, SMZ, and SP were higher than those for AGT, HLZ, INH, PZ, and PA (Figure 1F).

**TABLE 2:** Enzyme kinetic parameters for the *N*-acetylation of eight drugs using recombinant proteins encoded by NAT2*4, *5, *6, and *7 alleles.

	Km (μM)	Vmax (relative peak area/min/mg protein)	CLint (Vmax/Km)
AGT			
*4	76.24 ± 2.49	45.18 ± 1.52	0.593 ± 0.010
*5	104.07 ± 8.65	10.87 ± 0.65^a^	0.105 ± 0.004^a^
*6	187.75 ± 26.36^a,b^	6.70 ± 0.51^a,b^	0.037 ± 0.003^a,b^
*7	188.83 ± 5.56^a,b^	5.44 ± 0.18^a,b^	0.029 ± 0.001^a,b^
DDP			
*4	98.17 ± 8.60	24.73 ± 1.67	0.255 ± 0.018
*5	85.31 ± 10.69	4.86 ± 0.06^*a*^	0.059 ± 0.006^*a*^
*6	15.30 ± 0.44^a,b^	0.54 ± 0.03^a,b^	0.035 ± 0.001^a^
*7	5.21 ± 0.26^a,b^	0.96 ± 0.01^a,b^	0.185 ± 0.008^a,b,c^
HLZ			
*4	5.79 ± 0.26	1023.20 ± 33.02	177.09 ± 4.94
*5	4.68 ± 0.19^a^	224.78 ± 3.85^a^	48.15 ± 1.42^a^
*6	2.97 ± 0.17^a,b^	45.78 ± 1.65^a,b^	15.46 ± 0.41^a,b^
*7	4.76 ± 0.26^a,c^	28.29 ± 1.38^a,b^	5.95 ± 0.10^a,b^
INH^1^			
*4	128.10 ± 4.54	1496.50 ± 57.79	11.706 ± 0.422
*5	139.80 ± 16.33	196.35 ± 8.57^a^	1.449 ± 0.126^a^
*6	135.20 ± 9.81	61.94 ± 2.22^a,b^	0.462 ± 0.019^a,b^
*7	230.50 ± 22.29^a,b,c^	69.22 ± 2.00^a,b^	0.306 ± 0.021^a,b^
PZ			
*4	192.20 ± 11.70	67.02 ± 5.27	0.348 ± 0.012
*5	134.50 ± 11.59	13.27 ± 0.97^a^	0.102 ± 0.014^a^
*6	174.35 ± 23.60	7.30 ± 0.38^a^	0.044 ± 0.006^a,b^
*7	376.68 ± 28.77^a,b,c^	6.81 ± 0.07^a^	0.018 ± 0.001^a,b^
PA			
*4	193.13 ± 15.16	78.33 ± 2.19	0.410 ± 0.019
*5	164.65 ± 6.65	19.63 ± 0.28^a^	0.120 ± 0.006^a^
*6	220.30 ± 9.34^b^	17.04 ± 0.25^a^	0.078 ± 0.002^a^
*7	167.73 ± 4.22^c^	10.13 ± 0.24^a,b,c^	0.061 ± 0.002^a,b^
SMZ			
*4	86.24 ± 6.58	4.83 ± 0.17	0.056 ± 0.002
*5	136.00 ± 5.87^a^	0.98 ± 0.06^a^	0.007 ± 0.000^a^
*6	85.95 ± 2.92^b^	0.34 ± 0.00^a,b^	0.004 ± 0.001^a^
*7	12.37 ± 1.15^a,b,c^	0.21 ± 0.01^a,b^	0.017 ± 0.002^a,b,c^
SP			
*4	174.90 ± 20.79	63.53 ± 4.73	0.369 ± 0.019
*5	162.70 ± 12.93	13.44 ± 0.17^a^	0.084 ± 0.006^a^
*6	142.88 ± 14.10	6.18 ± 0.13^a^	0.044 ± 0.003^a^
*7	27.50 ± 1.10^a,b,c^	4.60 ± 0.07^a^	0.168 ± 0.010^a,b,c^

*Each value represents the mean ± SEM of four independent experiments. Tukey’s multiple comparison test; ^*a*^*P* < 0.05 compared with NAT2^∗^4, ^*b*^*P* < 0.05 compared with NAT2^∗^5, ^*c*^*P* < 0.05 compared with NAT2^∗^6. The data were reported previously (Suvichapanich et al., 2018).*

**FIGURE 2:**
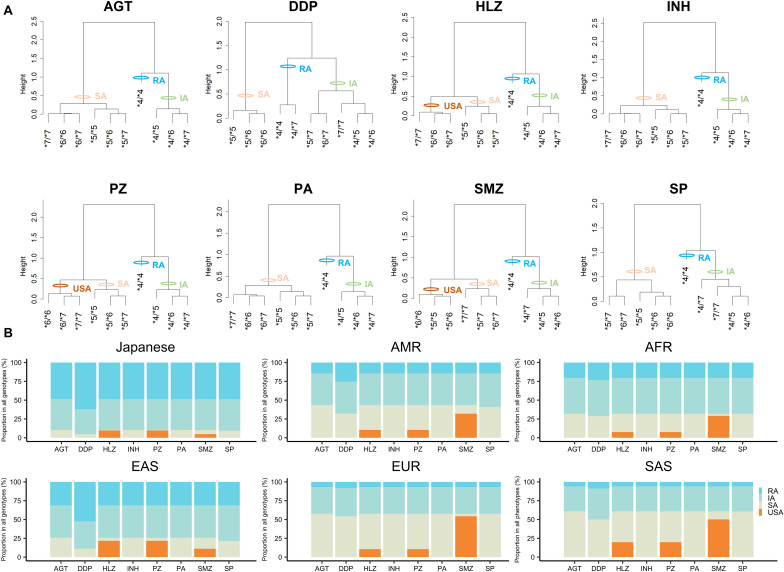
Categorization of *NAT2* genotypes into phenotypes by hierarchical cluster analysis based on activity scores and worldwide distribution of NAT2 phenotypes for different substrate drugs. **(A)** Cluster dendrograms using activity scores of *NAT2* genotypes for eight drugs and categorization of *NAT2* genotypes into the different phenotypes. **(B)** Proportion of predicted NAT2 phenotypes for each drug in Japanese, AFR, AMR, EAS, EUR, and SAS populations.

Using the CLint value of the RA allele, i.e., NAT2^∗^4 as a base of 1, we obtained activity scores for NAT2^∗^5, ^∗^6, and ^∗^7 according to the CLint values for different drugs (a value of 0.5 indicated a 50% reduction in the CLint). The sum of the activity scores of both alleles indicated the *NAT2* genotype for each drug (Table 3). For the classification of *NAT2* genotypes into phenotypes, such as RAs, IAs, SAs, and USAs, dendrograms were generated to visualize the relationships between activity scores and genotypes by hierarchical agglomerative clustering (Figure 2A). The most optimal number of clusters was three or four, according to analyses using NbClust R-packages. In the current study, 10 *NAT2* genotypes were classified as USAs, SAs, IAs, or RAs, based on the activity scores and results of clustering (Figure 2A and Table 3). The *NAT2^∗^4/^∗^4* genotype was categorized into the RA category for *N*-acetylation of all drugs. Ten *NAT2* genotypes were categorized into similar clusters for *N*-acetylation of AGT, HLZ, INH, PZ, and PA, whereas the numbers of clusters for AGT/INH/PA and HLZ/PZ were three and four, respectively. In cases of INH, all *NAT2* genotypes were divided into three phenotypes, i.e., SAs, IAs, and RAs, consistent with previous studies (Naidoo et al., 2019; Mthiyane et al., 2020). Owing to the higher activity scores and lower Km of the NAT2^∗^7 allele, the clustering patterns of 10 *NAT2* genotypes for *N*-acetylation of DDP, SMZ, and SP were different from those of other drugs. Although USAs were observed for *N*-acetylation of HLZ, PZ, and SMZ, the clustering patterns of 10 *NAT2* genotypes for SMZ were different from those of HLZ and PZ.

**TABLE 3:** Activity scores based on the values of intrinsic clearance and prediction of NAT2 phenotypes for eight drugs.

	AGT		DDP		HLZ		INH		PZ		PA		SMZ		SP	
Genotypes	AS	Predicted phenotype	AS	Predicted phenotype	AS	Predicted phenotype	AS	Predicted phenotype	AS	Predicted phenotype	AS	Predicted phenotype	AS	Predicted phenotype	AS	Predicted phenotype
*4/*4	2.000	RA	2.000	RA	2.000	RA	2.000	RA	2.000	RA	2.000	RA	2.000	RA	2.000	RA
*4/*5	1.178	IA	1.232	IA	1.272	IA	1.124	IA	1.315	IA	1.293	IA	1.129	IA	1.229	IA
*4/*6	1.062	IA	1.151	IA	1.088	IA	1.039	IA	1.140	IA	1.191	IA	1.071	IA	1.120	IA
*4/*7	1.049	IA	1.782	RA	1.034	IA	1.026	IA	1.058	IA	1.149	IA	1.304	IA	1.458	IA
*5/*5	0.356	SA	0.465	SA	0.544	SA	0.248	SA	0.630	SA	0.586	SA	0.258	USA	0.457	SA
*5/*6	0.240	SA	0.383	SA	0.360	SA	0.163	SA	0.455	SA	0.484	SA	0.200	USA	0.349	SA
*5/*7	0.226	SA	1.014	IA	0.306	SA	0.150	SA	0.373	SA	0.442	SA	0.433	SA	0.686	SA
*6/*6	0.125	SA	0.302	SA	0.175	USA	0.079	SA	0.279	USA	0.382	SA	0.142	USA	0.241	SA
*6/*7	0.111	SA	0.933	IA	0.121	USA	0.066	SA	0.198	USA	0.340	SA	0.374	SA	0.578	SA
*7/*7	0.097	SA	1.564	RA	0.067	USA	0.052	SA	0.116	USA	0.298	SA	0.607	SA	0.916	IA

*AS, activity score; IA, intermediate acetylator; RA, rapid acethylator; SA, slow acetylator; USA, ultra-slow acetylator.*

The genetic distribution of the predicted NAT2 phenotypes based on the activity scores of Japanese, AFR, AMR, EAS, EUR, and SAS populations in the 1KGP are presented in Figure 2B and Supplementary Figure 1. RAs for NAT2 were present at high frequencies, particularly in Japanese (48.48-62.02%) and EAS (31.15-52.38%) populations, but were present at low frequencies in EUR (6.76-7.95%) and SAS (5.93-8.79%) populations. According to the results of *in vitro* metabolic experiments using SMZ as a substrate, the frequencies of USAs were much higher (29.05-54.27%) than those of other drugs, except in Japanese and EAS populations (4.75 and 11.11%, respectively).

## Discussion

*NAT2* genotypes show marked geographic and ethnic differences. In order to clarify the substrate-specific diversity of NAT2 phenotypes between different populations, we categorized *NAT2* genotypes into USA, SA, IA, and RA phenotypes in 26 populations based on the results of *in vitro N*-acetylation of eight drugs. Our analyses revealed the dramatic genetic variability between populations, including phenotypic consequences at the level of *N*-acetylation profiles. In particular, we observed lower frequencies of RAs of *N*-acetylation for all substrates in EUR and SAS populations, which showed normal NAT2 activity, suggesting that lower dosages of NAT2 substrate drugs in EUR and SAS populations may be more appropriate than a one-size-fits-all approach. Thus, our findings provide useful information for population-adjusted genotype-guided therapy for NAT2.

The worldwide distribution of NAT2 phenotype diversity, i.e., SAs, IAs, and RAs, has been reported (Walker et al., 2009; Sabbagh et al., 2011), and genetic differentiation patterns have been shown to be related to geography (Sabbagh et al., 2008). However, no reports have described the diversity of NAT2 phenotypes for USAs, which were recently identified based on combined *^∗^6/^∗^6*, *^∗^6/^∗^7*, and *^∗^7/^∗^7* genotypes (Selinski et al., 2013; Selinski et al., 2015; Suarez-Kurtz et al., 2016). Our study showed that genotypes could be categorized into the USA phenotype in *N*-acetylation of HLZ, PZ, and SMZ by hierarchical clustering analysis. In a previous work, the pharmacokinetics of oral HLZ were found to be dependent on the *NAT2* genotype during pregnancy (Han et al., 2019), and SA status was shown to be associated with clinical blood pressure and 24-h blood pressure after HLZ treatment in patients with resistant hypertension (Garces-Eisele et al., 2014). Therefore, the *^∗^6/^∗^6*, *^∗^6/^∗^7*, and *^∗^7/^∗^7* genotypes must be clearly distinct from the SA group, and this categorization may further improve individualization of HLZ treatment.

In this study, we focused only on alleles (*^∗^5*, *^∗^6*, and *^∗^7*) with frequencies equal to 5% or higher in 2,504 individuals of the 1KG project. Although the minor allele frequency (MAF) of the rs1801279 defining *NAT2^∗^14* (which confers an SA phenotype) was 2.78% in all individuals of the 1KG project, the African subpopulations show MAFs higher than 5%. Indeed, the MAFs of ACB, ASW, ESN, GWD, LWK, MSL, and YRI populations in Africa were 7.8, 7.4, 12.6, 14.6, 9.1, 7.1, and 11.6%, respectively. In the seven African subpopulations, the non-synonymous variants defining *NAT2^∗^22* (0.98%) and *^∗^24* (2.34%) were also detected. Therefore, the frequency of SA in the African population in our study may be underestimated. Further studies focusing on *NAT2^∗^14, ^∗^22, ^∗^24* and other rare alleles are needed.

In previous *in vitro* metabolic experiments using SMZ as a substrate, NAT2^∗^5, ^∗^6, and ^∗^7 showed lower Vmax values than that of NAT2^∗^4, but only NAT2^∗^7 showed higher affinity for SMZ with lower Km compared with NAT2^∗^4 (Olivera et al., 2007; Garces-Eisele et al., 2014), consistent with our current study. Additionally, NAT2*^∗^5* and *^∗^6* alleles result in lower *N*-acetyltransferase activities toward SMZ compared with NAT2*^∗^7* (Walraven et al., 2008), and carriers of *NAT2^∗^5* and *^∗^6* were categorized as USAs in the current study. Moreover, the NAT2 SA phenotype is associated with the pharmacokinetics of a different sulfur drug, sulfamethoxazole, in renal transplant recipients (Kagaya et al., 2012) and with adverse reactions to sulfamethoxazole, such as toxic epidermal necrolysis, Stevens-Johnson syndrome, and increased serum alanine aminotransferase levels in patients with systemic lupus erythematosus (Soejima et al., 2007). For SMZ and structurally related drugs, such as sulfamethoxazole, the categorization of carriers of *NAT2^∗^5* and *^∗^6* alleles as USAs may be useful for genotype-guided dosing.

In summary, in this study, we defined NAT2 phenotypes based on the activity score of each drug and determined the worldwide distribution of the NAT2 phenotype diversity according to this new categorization method. Because limited information on *NAT2* genotypes has been published, our current frequency data for the large-scale Japanese population and 26 different populations collected by the 1KGP should be valuable. Therefore, our findings will be useful for future studies, including case-control association studies, to predict responses to drugs primarily metabolized by NAT2. To verify the findings of the present study, in the future, case-control association studies to predict the risk of adverse drug reaction and drug responses should be conducted. By the verification of the benefit of usage of information on the NAT2 phenotypes depending on each drug and the different populations, we will be able to implement the NAT2 phenotypes as pharmacogenomics biomarkers.

## Data Availability Statement

The datasets presented in this study can be found in online repositories. The names of the repository/repositories and accession number(s) can be found in the article/ Supplementary Material.

## Ethics Statement

The studies involving human participants were reviewed and approved by the study was approved by the Ethics Committee of National Cancer Center Research Institute, Fujita Health University Hospital, and RIKEN Center for Integrative Medical Sciences. The patients/participants provided their written informed consent to participate in this study.

## Author Contributions

KF and TM conceived the study and designed the experiments. KK, TO, TY, TS, HZ, MI, and NI supplied the all genomic DNAs. KF performed the experiments, analyzed the data, and wrote the manuscript. All authors reviewed the manuscript and approved the final version to be published.

## Conflict of Interest

The authors declare that the research was conducted in the absence of any commercial or financial relationships that could be construed as a potential conflict of interest. The handling editor declared a past co-authorship with several of the authors KF and TM.

## Footnotes

1 http://nat.mbg.duth.gr/

2 https://humandbs.biosciencedbc.jp/hum0163-v2

3 http://ftp.1000genomes.ebi.ac.uk/vol1/ftp/release/20130502/

## References

[B1] An Z. Li C. Lv Y. Li P. Wu C. Liu L. (2018). Metabolomics of hydrazine-induced hepatotoxicity in rats for discovering potential biomarkers. *Dis. Mark.* 2018:8473161. 10.1155/2018/8473161 PMC5914126 29849827

[B2] Azuma J. Ohno M. Kubota R. Yokota S. Nagai T. Tsuyuguchi K. (2013). NAT2 genotype guided regimen reduces isoniazid-induced liver injury and early treatment failure in the 6-month four-drug standard treatment of tuberculosis: a randomized controlled trial for pharmacogenetics-based the rapy. *Eur. J. Clin. Pharmacol.* 69 1091–1101. 10.1007/s00228-012-1429-9 23150149 PMC3641305

[B3] Birch Kristensen E. Yakimov V. Bjorn-Mortensen K. Soborg B. Koch A. Andersson M. (2018). Study of correlation between the NAT2 phenotype and genotype status among greenlandic inuit. *EXCLI J.* 17 1043–1053. 30564082 10.17179/excli2018-1671 PMC6295636

[B4] Brewer C. T. Yang L. Edwards A. Lu Y. Low J. Wu J. (2019). The isoniazid metabolites hydrazine and pyridoxal isonicotinoyl hydrazone modulate heme biosynthesis. *Toxicol. Sci.* 168 209–224. 10.1093/toxsci/kfy294 30517741 PMC6390808

[B5] Fukunaga K. Hishinuma E. Hiratsuka M. Kato K. Okusaka T. Saito T. (2020). Determination of novel CYP2D6 haplotype using the targeted sequencing followed by the long-read sequencing and the functional characterization in the Japanese population. *J. Hum. Genet.* 66 139–149. 10.1038/s10038-020-0815-x 32759992

[B6] Garces-Eisele S. J. Cedillo-Carvallo B. Reyes-Nunez V. Estrada-Marin L. Vazquez-Perez R. Juarez-Calderon M. (2014). Genetic selection of volunteers and concomitant dose adjustment leads to comparable hydralazine/valproate exposure. *J. Clin. Pharm. Ther.* 39 368–375. 10.1111/jcpt.12155 24702251

[B7] Han L. W. Ryu R. J. Cusumano M. Easterling T. R. Phillips B. R. Risler L. J. (2019). Effect of N-Acetyltransferase 2 genotype on the pharmacokinetics of hydralazine during pregnancy. *J. Clin. Pharmacol.* 59 1678–1689. 10.1002/jcph.1477 31257615 PMC6813860

[B8] Hein D. W. Boukouvala S. Grant D. M. Minchin R. F. Sim E. (2008). Changes in consensus arylamine N-acetyltransferase gene nomenclature. *Pharmacogenet. Genomics* 18 367–368. 10.1097/fpc.0b013e3282f60db0 18334921 PMC2386960

[B9] Kagaya H. Miura M. Niioka T. Saito M. Numakura K. Habuchi T. (2012). Influence of NAT2 polymorphisms on sulfamethoxazole pharmacokinetics in renal transplant recipients. *Antimicrob. Agents Chemother.* 56 825–829. 10.1128/aac.05037-11 22106207 PMC3264276

[B10] Li J. Zhang L. Zhou H. Stoneking M. Tang K. (2011). Global patterns of genetic diversity and signals of natural selection for human ADME genes. *Hum. Mol. Genet.* 20 528–540. 10.1093/hmg/ddq498 21081654

[B11] Mortensen H. M. Froment A. Lema G. Bodo J. M. Ibrahim M. Nyambo T. B. (2011). Characterization of genetic variation and natural selection at the arylamine N-acetyltransferase genes in global human populations. *Pharmacogenomics* 12 1545–1558. 10.2217/pgs.11.88 21995608 PMC4653814

[B12] Mthiyane T. Millard J. Adamson J. Balakrishna Y. Connolly C. Owen A. (2020). N-Acetyltransferase 2 genotypes among zulu-speaking South Africans and Isoniazid and N-Acetyl-Isoniazid Pharmacokinetics during Antituberculosis treatment. *Antimicrob. Agents Chemother.* 64:e02376-19. 10.1128/AAC.02376-19 PMC7179278 31964788

[B13] Mushiroda T. Yanai H. Yoshiyama T. Sasaki Y. Okumura M. Ogata H. (2016). Development of a prediction system for anti-tuberculosis drug-induced liver injury in Japanese patients. *Hum. Genome Var.* 3:16014. 10.1038/hgv.2016.14 PMC4917605 27340556

[B14] Naidoo A. Chirehwa M. Ramsuran V. Mcilleron H. Naidoo K. Yende-Zuma N. (2019). Effects of genetic variability on rifampicin and isoniazid pharmacokinetics in South African patients with recurrent tuberculosis. *Pharmacogenomics* 20 225–240. 10.2217/pgs-2018-0166 30767706 PMC6562923

[B15] Nicoletti P. Devarbhavi H. Goel A. Venkatesan R. Eapen C. E. Grove J. I. (2020). Genetic risk factors in drug-induced liver injury due to isoniazid-containing antituberculosis drug regimens. *Clin. Pharmacol. Ther.* 10.1002/cpt.2100 Online ahead of print. 33135175

[B16] Olivera M. Martinez C. Gervasini G. Carrillo J. A. Ramos S. Benitez J. (2007). Effect of common NAT2 variant alleles in the acetylation of the major clonazepam metabolite, 7-aminoclonazepam. *Drug Metab. Lett.* 1 3–5. 10.2174/187231207779814283 19356010

[B17] Rens N. E. Uyl-De Groot C. A. Goldhaber-Fiebert J. D. Croda J. Andrews J. R. (2020). Cost-effectiveness of a pharmacogenomic test for stratified isoniazid dosing in treatment of active tuberculosis. *Clin. Infect. Dis.* 71 3136–3143. 10.1093/cid/ciz1212 31905381 PMC7819527

[B18] Ruiz J. D. Martinez C. Anderson K. Gross M. Lang N. P. Garcia-Martin E. (2012). The differential effect of NAT2 variant alleles permits refinement in phenotype inference and identifies a very slow acetylation genotype. *PLoS One* 7:e44629. 10.1371/journal.pone.0044629 22970273 PMC3435299

[B19] Sabbagh A. Darlu P. Crouau-Roy B. Poloni E. S. (2011). Arylamine N-acetyltransferase 2 (NAT2) genetic diversity and traditional subsistence: a worldwide population survey. *PLoS One* 6:e18507. 10.1371/journal.pone.0018507 21494681 PMC3071824

[B20] Sabbagh A. Langaney A. Darlu P. Gerard N. Krishnamoorthy R. Poloni E. S. (2008). Worldwide distribution of NAT2 diversity: implications for NAT2 evolutionary history. *BMC Genet.* 9:21. 10.1186/1471-2156-9-21 PMC2292740 18304320

[B21] Selinski S. Blaszkewicz M. Ickstadt K. Hengstler J. G. Golka K. (2013). Refinement of the prediction of N-acetyltransferase 2 (NAT2) phenotypes with respect to enzyme activity and urinary bladder cancer risk. *Arch. Toxicol.* 87 2129–2139. 10.1007/s00204-013-1157-7 24221535

[B22] Selinski S. Getzmann S. Gajewski P. D. Blaszkewicz M. Hengstler J. G. Falkenstein M. (2015). The ultra-slow NAT2^∗^6A haplotype is associated with reduced higher cognitive functions in an elderly study group. *Arch. Toxicol.* 89 2291–2303. 10.1007/s00204-015-1635-1 26615528

[B23] Selinski S. Gerullis H. Otto T. Roth E. Volkert F. Ovsiannikov D. (2017). Ultra-slow N-Acetyltransferase 2 is associated with recurrence-free time in bladder cancer patients. *Eur. Urol.* 71 994–995. 10.1016/j.eururo.2016.12.007 28040354

[B24] Soejima M. Sugiura T. Kawaguchi Y. Kawamoto M. Katsumata Y. Takagi K. (2007). Association of the diplotype configuration at the N-acetyltransferase 2 gene with adverse events with co-trimoxazole in Japanese patients with systemic lupus erythematosus. *Arthritis Res. Ther.* 9:R23. 10.1186/ar2134 PMC1906798 17335581

[B25] Spinasse L. B. Santos A. R. Suffys P. N. Muxfeldt E. S. Salles G. F. (2014). Different phenotypes of the NAT2 gene influences hydralazine antihypertensive response in patients with resistant hypertension. *Pharmacogenomics* 15 169–178. 10.2217/pgs.13.202 24444407

[B26] Suarez-Kurtz G. Fuchshuber-Moraes M. Struchiner C. J. Parra E. J. (2016). Single nucleotide polymorphism coverage and inference of N-acetyltransferase-2 acetylator phenotypes in wordwide population groups. *Pharmacogenet. Genomics.* 26 363–369. 10.1097/fpc.0000000000000225 27136043

[B27] Suvichapanich S. Fukunaga K. Zahroh H. Mushiroda T. Mahasirimongkol S. Toyo-Oka L. (2018). NAT2 ultra-slow acetylator and risk of anti-tuberculosis drug-induced liver injury: a genotype-based meta-analysis. *Pharmacogenet. Genomics.* 28 167–176. 10.1097/fpc.0000000000000339 29781872

[B28] Suvichapanich S. Wattanapokayakit S. Mushiroda T. Yanai H. Chuchottawon C. Kantima T. (2019). Genomewide association study confirming the association of NAT2 with susceptibility to antituberculosis drug-induced liver injury in thai patients. *Antimicrob. Agents Chemother.* 63:e02692-18. 10.1128/AAC.02692-18 PMC6658740 31109976

[B29] Walker K. Ginsberg G. Hattis D. Johns D. O. Guyton K. Z. Sonawane B. (2009). Genetic polymorphism in N-Acetyltransferase (NAT): population distribution of NAT1 and NAT2 activity. *J. Toxicol. Environ. Health B. Crit. Rev.* 12 440–472. 10.1080/10937400903158383 20183529

[B30] Walraven J. M. Zang Y. Trent J. O. Hein D. W. (2008). Structure/function evaluations of single nucleotide polymorphisms in human N-acetyltransferase 2. *Curr. Drug Metab.* 9 471–486. 10.2174/138920008784892065 18680467 PMC2507886

[B31] Wattanapokayakit S. Mushiroda T. Yanai H. Wichukchinda N. Chuchottawon C. Nedsuwan S. (2016). NAT2 slow acetylator associated with anti-tuberculosis drug-induced liver injury in Thai patients. *Int. J. Tuberc. Lung Dis.* 20 1364–1369. 10.5588/ijtld.15.0310 27725049

[B32] Yuliwulandari R. Prayuni K. Razari I. Susilowati R. W. Zulhamidah Y. Soedarsono S. (2021). Genetic characterization of N-acetyltransferase 2 variants in acquired multidrug-resistant tuberculosis in Indonesia. *Pharmacogenomics* 10.2217/pgs-2020-0163 Online ahead of print. 33399479

[B33] Yuliwulandari R. Susilowati R. W. Wicaksono B. D. Viyati K. Prayuni K. Razari I. (2016). NAT2 variants are associated with drug-induced liver injury caused by anti-tuberculosis drugs in Indonesian patients with tuberculosis. *J. Hum. Genet.* 61 533–537. 10.1038/jhg.2016.10 26911349

[B34] Zang Y. Doll M. A. Zhao S. States J. C. Hein D. W. (2007). Functional characterization of single-nucleotide polymorphisms and haplotypes of human N-acetyltransferase 2. *Carcinogenesis* 28 1665–1671. 10.1093/carcin/bgm085 17434923 PMC2085371

