# Supplementary material for: Functional Characterization of the Effects of N-acetyltransferase 2 Alleles on N-acetylation of Eight Drugs and Worldwide Distribution of Substrate-Specific Diversity
Source: Front Genet. 2021 Mar 18;12:652704. doi: 10.3389/fgene.2021.652704 (PMC8012690; doi:10.3389/fgene.2021.652704)
Supplement: Supplementary file 1 [file Data_Sheet_1.docx]

Supplementary Material

# Supplementary Figures


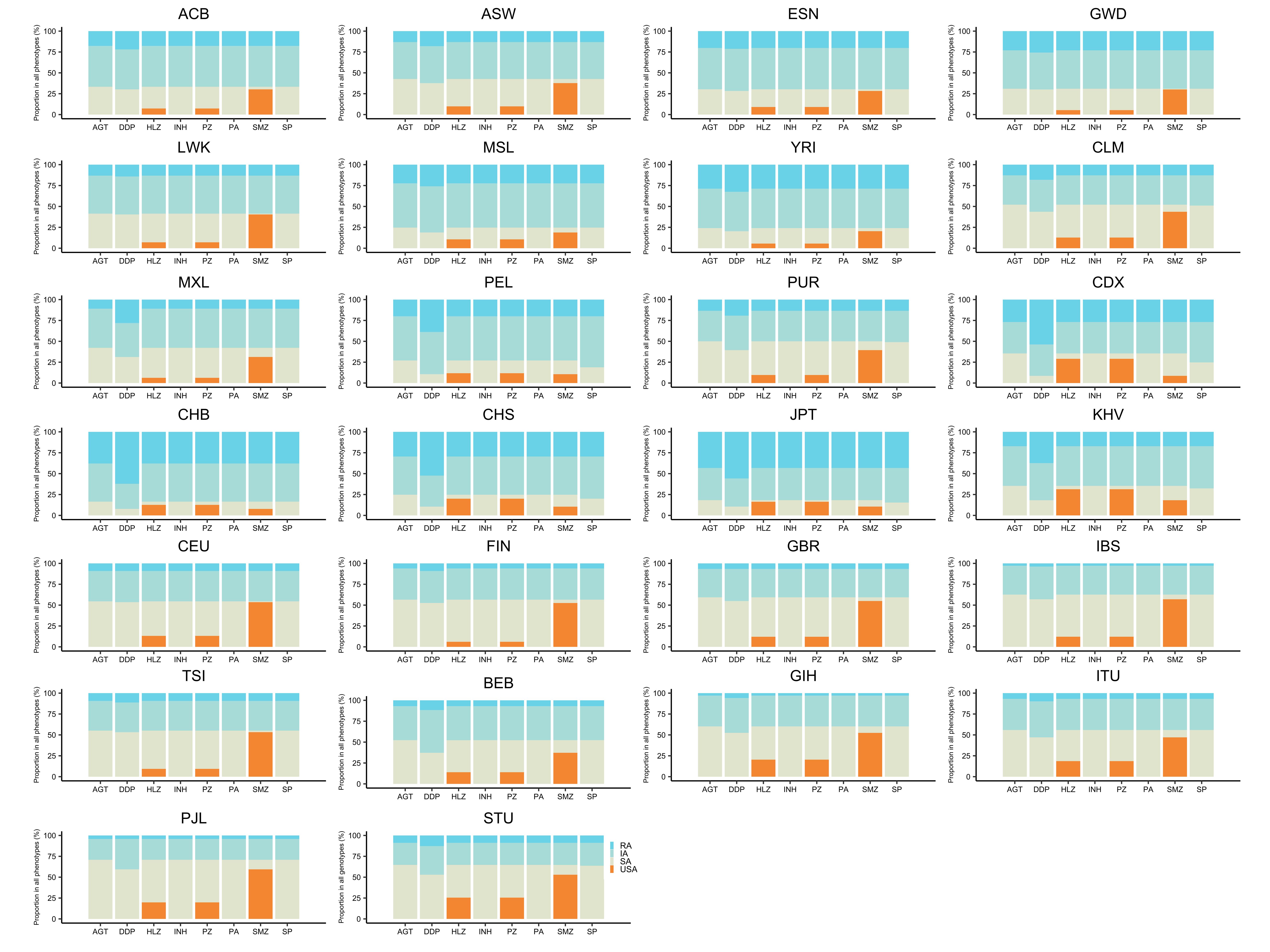


**Supplementary Figure 1.** **Worldwide distribution of NAT2 phenotypes for different substrate drugs in 26 populations.** Proportion of the predicted NAT2 phenotype for each drug in 26 populations of the 1KG Genome Project. African Caribbeans in Barbados (ACB); Americans of African ancestry in southwest USA (ASW); Esan in Nigeria (ESN); Luhya in Webuye, Kenya (LWK); Gambian in Western Divisions in the Gambia (GWD); Mende in Sierra Leone (MSL); and Yoruba in Ibadan, Nigeria (YRI). The AMR population consisted of Colombians from Medellin, Colombia (CLM); Mexicans from Los Angeles, USA (MXL); Peruvians from Lima, Peru (PEL); and Puerto Ricans from Puerto Rico (PUR). The EAS population consisted of Chinese Dai in Xishuangbanna, China (CDX); Han Chinese in Beijing, China (CHB); Southern Han Chinese (CHS); Japanese in Tokyo, Japan (JPT); and Kinh in Ho Chi Minh City, Vietnam (KHV). The EUR population consisted of Utah residents with northern and western European ancestry (CEU), Finnish in Finland (FIN), British in England and Scotland (GBR), Iberians in Spain (IBS); and Toscani in Italy (TSI). The SAS population consisted of Bengali from Bangladesh (BEB); Gujarati Indians from Houston, TX, USA (GIH); Indian Telugu from the UK (ITU); Punjabis from Lahore, Pakistan (PJL); and Sri Lankan Tamils from the UK (STU). Eight drugs (aminoglutethimide, AGT; diaminodiphenyl sulfone, DDP; hydralazine, HLZ; isoniazid, INH; phenelzine, PZ; procaineamide, PA; sulfamethazine, SMZ; and sulfapyrizine, SP) were used as NAT2 substrates.

# Supplementary tables

**Table S1. Frequency of *NAT2* genotypes in 2504 individuals of 26 populations of 1KG.**

| Population Code | **4/*4* | **4/*5* | **4/*6* | **4/*7* | **5/*5* | **5/*6* | **5/*7* | **6/*6* | **6/*7* | **7/*7* |
| --- | --- | --- | --- | --- | --- | --- | --- | --- | --- | --- |
| ACB | 0.1771 | 0.2083 | 0.2396 | 0.0417 | 0.0938 | 0.1458 | 0.0208 | 0.0625 | 0.0104 | 0.0000 |
| ASW | 0.1311 | 0.1803 | 0.2131 | 0.0492 | 0.1148 | 0.2131 | 0.0000 | 0.0492 | 0.0492 | 0.0000 |
| ESN | 0.2020 | 0.2626 | 0.2222 | 0.0101 | 0.0707 | 0.1313 | 0.0101 | 0.0808 | 0.0101 | 0.0000 |
| GWD | 0.2301 | 0.2832 | 0.1504 | 0.0265 | 0.1327 | 0.1239 | 0.0000 | 0.0442 | 0.0088 | 0.0000 |
| LWK | 0.1313 | 0.2626 | 0.1818 | 0.0101 | 0.1111 | 0.2323 | 0.0000 | 0.0606 | 0.0101 | 0.0000 |
| MSL | 0.2235 | 0.2824 | 0.2118 | 0.0353 | 0.0588 | 0.0588 | 0.0235 | 0.0706 | 0.0353 | 0.0000 |
| YRI | 0.2870 | 0.2222 | 0.2130 | 0.0370 | 0.0741 | 0.0926 | 0.0185 | 0.0370 | 0.0185 | 0.0000 |
| CLM | 0.1277 | 0.2021 | 0.1064 | 0.0426 | 0.1809 | 0.1809 | 0.0319 | 0.0745 | 0.0426 | 0.0106 |
| MXL | 0.1094 | 0.2344 | 0.0625 | 0.1719 | 0.1719 | 0.1250 | 0.0625 | 0.0156 | 0.0469 | 0.0000 |
| PEL | 0.2000 | 0.3529 | 0.0706 | 0.1059 | 0.0471 | 0.0588 | 0.0471 | 0.0000 | 0.0353 | 0.0824 |
| PUR | 0.1346 | 0.1923 | 0.1250 | 0.0481 | 0.1923 | 0.1538 | 0.0577 | 0.0481 | 0.0385 | 0.0096 |
| CDX | 0.2688 | 0.0323 | 0.1828 | 0.1613 | 0.0000 | 0.0323 | 0.0323 | 0.0538 | 0.1290 | 0.1075 |
| CHB | 0.3786 | 0.0194 | 0.1942 | 0.2427 | 0.0000 | 0.0291 | 0.0097 | 0.0485 | 0.0777 | 0.0000 |
| CHS | 0.2952 | 0.0286 | 0.2476 | 0.1810 | 0.0000 | 0.0476 | 0.0000 | 0.0571 | 0.0952 | 0.0476 |
| JPT | 0.4327 | 0.0192 | 0.2692 | 0.0962 | 0.0000 | 0.0192 | 0.0000 | 0.0865 | 0.0481 | 0.0288 |
| KHV | 0.1717 | 0.0606 | 0.2424 | 0.1717 | 0.0101 | 0.0202 | 0.0101 | 0.1515 | 0.1313 | 0.0303 |
| CEU | 0.0909 | 0.2424 | 0.1212 | 0.0000 | 0.1919 | 0.2121 | 0.0101 | 0.1313 | 0.0000 | 0.0000 |
| FIN | 0.0606 | 0.1818 | 0.1616 | 0.0303 | 0.2222 | 0.2626 | 0.0202 | 0.0404 | 0.0202 | 0.0000 |
| GBR | 0.0659 | 0.2198 | 0.1209 | 0.0000 | 0.2418 | 0.1868 | 0.0440 | 0.1209 | 0.0000 | 0.0000 |
| IBS | 0.0280 | 0.2150 | 0.1215 | 0.0093 | 0.2243 | 0.2430 | 0.0374 | 0.1028 | 0.0187 | 0.0000 |
| TSI | 0.0935 | 0.2056 | 0.1308 | 0.0187 | 0.1963 | 0.2523 | 0.0093 | 0.0841 | 0.0093 | 0.0000 |
| BEB | 0.0698 | 0.1860 | 0.1744 | 0.0465 | 0.1512 | 0.1512 | 0.0814 | 0.0698 | 0.0698 | 0.0000 |
| GIH | 0.0291 | 0.1456 | 0.1942 | 0.0291 | 0.1456 | 0.2136 | 0.0388 | 0.1650 | 0.0388 | 0.0000 |
| ITU | 0.0686 | 0.1961 | 0.1471 | 0.0294 | 0.1176 | 0.2255 | 0.0294 | 0.1275 | 0.0588 | 0.0000 |
| PJL | 0.0417 | 0.1458 | 0.1042 | 0.0000 | 0.1667 | 0.3021 | 0.0417 | 0.1250 | 0.0729 | 0.0000 |
| STU | 0.0882 | 0.0980 | 0.1373 | 0.0294 | 0.0686 | 0.2941 | 0.0294 | 0.1667 | 0.0784 | 0.0098 |

African Caribbeans in Barbados (ACB); Americans of African ancestry in southwest USA (ASW); Esan in Nigeria (ESN); Luhya in Webuye, Kenya (LWK); Gambian in Western Divisions in the Gambia (GWD); Mende in Sierra Leone (MSL); and Yoruba in Ibadan, Nigeria (YRI). The AMR population consisted of Colombians from Medellin, Colombia (CLM); Mexicans from Los Angeles, USA (MXL); Peruvians from Lima, Peru (PEL); and Puerto Ricans from Puerto Rico (PUR). The EAS population consisted of Chinese Dai in Xishuangbanna, China (CDX); Han Chinese in Beijing, China (CHB); Southern Han Chinese (CHS); Japanese in Tokyo, Japan (JPT); and Kinh in Ho Chi Minh City, Vietnam (KHV). The EUR population consisted of Utah residents with northern and western European ancestry (CEU), Finnish in Finland (FIN), British in England and Scotland (GBR), Iberians in Spain (IBS); and Toscani in Italy (TSI). The SAS population consisted of Bengali from Bangladesh (BEB); Gujarati Indians from Houston, TX, USA (GIH); Indian Telugu from the UK (ITU); Punjabis from Lahore, Pakistan (PJL); and Sri Lankan Tamils from the UK (STU).

**Table S2. *P* values of the results of Tukey's multiple comparison tests for the eight drugs using the relative clearance values of NAT2*5, *6, and *7.**

|  | P values |  |  |
| --- | --- | --- | --- |
|  | *5 | *6 | *7 |
| AGT vs DDP | 0.5026 | 1.38E-05 | 2.11E-14 |
| AGT vs HLZ | 0.0377 | 0.3791 | 0.9988 |
| AGT vs INH | 0.5266 | 0.4950 | 0.9857 |
| AGT vs PZ | 0.0067 | 1.28E-09 | 0.0146 |
| AGT vs PA | 0.0067 | 0.0002 | 1.0000 |
| AGT vs SMZ | 0.6211 | 0.9943 | 1.52E-08 |
| AGT vs SP | 0.6064 | 0.0008 | 8.46E-13 |
| DDP vs HLZ | 0.8287 | 0.0029 | 2.10E-14 |
| DDP vs INH | 0.0115 | 1.57E-07 | 2.10E-14 |
| DDP vs PZ | 0.3927 | 0.0033 | 2.13E-14 |
| DDP vs PA | 0.3917 | 0.9681 | 2.11E-14 |
| DDP vs SMZ | 0.0165 | 0.0001 | 4.33E-13 |
| DDP vs SP | 1.0000 | 0.7055 | 5.51E-09 |
| HLZ vs INH | 0.0003 | 0.0061 | 1.0000 |
| HLZ vs PZ | 0.9944 | 9.15E-08 | 0.0036 |
| HLZ vs PA | 0.9943 | 0.0317 | 0.9944 |
| HLZ vs SMZ | 0.0005 | 0.8182 | 4.94E-09 |
| HLZ vs SP | 0.7400 | 0.1259 | 4.02E-13 |
| INH vs PZ | 0.0001 | 4.17E-11 | 0.0018 |
| INH vs PA | 0.0001 | 1.40E-06 | 0.9636 |
| INH vs SMZ | 1.0000 | 0.1527 | 2.87E-09 |
| INH vs SP | 0.0171 | 6.25E-06 | 2.83E-13 |
| PZ vs PA | 1.0000 | 0.0003 | 0.0212 |
| PZ vs SMZ | 0.0001 | 0.0010 | 2.09E-08 |
| PZ vs SP | 0.3055 | 0.9974 | 1.05E-12 |
| PA vs SMZ | 0.0001 | 5.07E-09 | 0.0001 |
| PA vs SP | 0.3063 | 0.0001 | 3.10E-10 |
| SMZ vs SP | 0.0242 | 0.0048 | 0.0001 |

Abbreviations: aminoglutethimide, AGT; diaminodiphenyl sulfone, DDP; hydralazine, HLZ; isoniazid, INH; phenelzine, PZ; procaineamide, PA; sulfamethazine, SMZ; and sulfapyrizine, SP.
